# Air Pollution and Lymphocyte Phenotype Proportions in Cord Blood

**DOI:** 10.1289/ehp.7610

**Published:** 2005-06-15

**Authors:** Irva Hertz-Picciotto, Caroline E.W. Herr, Poh-Sin Yap, Miroslav Dostál, Robert H. Shumway, Paul Ashwood, Michael Lipsett, Jesse P. Joad, Kent E. Pinkerton, Radim J. Šrám

**Affiliations:** 1 Department of Public Health Sciences, University of California, Davis, California, USA; 2 Institute of Hygiene and Environmental Medicine, University of Giessen, Germany; 3 Laboratory of Genetic Ecotoxicology, Institute of Experimental Medicine, Academy of Sciences of the Czech Republic, and Health Institute of Central Bohemia, Prague, Czech Republic; 4 Department of Statistics and; 5 Department of Rheumatology, Allergy, and Clinical Immunology, University of California, Davis, California, USA; 6 Department of Epidemiology and Biostatistics, University of California, San Francisco, California, USA; 7 Department of Pediatrics, and; 8 Center for Health and the Environment, University of California, Davis, California, USA

**Keywords:** air pollution, B-cell, cord blood, immune status, immunology, lymphocytes, natural killer cells, PAH, PM_10_, pregnancy, T-cell

## Abstract

Effects of air pollution on morbidity and mortality may be mediated by alterations in immune competence. In this study we examined short-term associations of air pollution exposures with lymphocyte immunophenotypes in cord blood among 1,397 deliveries in two districts of the Czech Republic. We measured fine particulate matter < 2.5 μm in diameter (PM_2.5_) and 12 polycyclic aromatic hydrocarbons (PAHs) in 24-hr samples collected by versatile air pollution samplers. Cord blood samples were analyzed using a FACSort flow cytometer to determine phenotypes of CD3^+^ T-lymphocytes and their subsets CD4^+^ and CD8^+^, CD19^+^ B-lymphocytes, and natural killer cells. The mothers were interviewed regarding sociodemographic and lifestyle factors, and medical records were abstracted for obstetric, labor and delivery characteristics. During the period 1994 to 1998, the mean daily ambient concentration of PM_2.5_ was 24.8 μg/m^3^ and that of PAHs was 63.5 ng/m^3^. In multiple linear regression models adjusted for temperature, season, and other covariates, average PAH or PM_2.5_ levels during the 14 days before birth were associated with decreases in T-lymphocyte phenotype fractions (i.e., CD3^+^ CD4^+^, and CD8^+^), and a clear increase in the B-lymphocyte (CD19^+^) fraction. For a 100-ng/m^3^ increase in PAHs, which represented approximately two standard deviations, the percentage decrease was −3.3% [95% confidence interval (CI), −5.6 to −1.0%] for CD3^+^, −3.1% (95% CI, −4.9 to −1.3%) for CD4^+^, and −1.0% (95% CI, −1.8 to −0.2%) for CD8^+^ cells. The corresponding increase in the CD19^+^ cell proportion was 1.7% (95% CI, 0.4 to 3.0%). Associations were similar but slightly weaker for PM_2.5_. Ambient air pollution may influence the relative distribution of lymphocyte immunophenotypes of the fetus.

Early life is a potentially susceptible period for pollution-induced perturbation of the respiratory system (Braun-Fahrländer et al. 1997), DNA (Binková et al. 1995), and possibly the immune system (Hertz-Picciotto et al. 2002). Disturbances during this developmental period may result in transient or irreversible long-term effects and may also provide models for examining the mechanisms by which air pollution may affect the entire population.

With regard to prenatal exposures, current evidence from several countries is compatible with small adverse effects of particulate matter (PM), polycyclic aromatic hydrocarbons (PAHs), and/or other air pollutants on fetal growth, infant mortality, and duration of pregnancy (Dejmek et al. 1999, 2000; Glinianaia et al. 2004; Jedrychowski et al. 2004; Perera et al. 2003; Šrám et al. 1999; Wang et al. 1997; Wilhelm and Ritz 2003). Ambient PAH exposure has also been linked to somatic mutations in newborns (Perera et al. 2002) and possibly to heritable genetic changes (Somers et al. 2004). Benzo[*a*]pyrene (BaP)–DNA adducts were found to be substantially higher in cord blood compared with maternal blood samples from New York City residents (Perera et al. 2004).

A recent European study reported associations of PM with absolute numbers of B-cells, CD4^+^ and CD8^+^ T-cells, and natural killer (NK) cells in schoolchildren, after adjustment for numerous factors (Leonardi et al. 2000). In earlier work, we observed lower percentages of T-lymphocyte and higher NK cell percentages among residents of a polluted community, and among deliveries in winter, when air pollutant levels are highest (Hertz-Picciotto et al. 2002). Ambient PM pollution causes oxidative stress (Li et al. 2003) and may influence allergic (Diaz-Sanchez et al. 2003), immunologic (Burchiel et al. 2004), and systemic inflammatory responses (Li et al. 2003; van Eeden and Hogg 2002).

A further rationale for concern about ambient air pollution is the similarity with constituents of cigarette smoke. Evidence of early-life vulnerability to cigarette smoke is seen in reduced physical height; higher incidence of lower respiratory infections, asthma and wheeze, middle-ear disease, and sudden infant death syndrome (SIDS) (Courage 2002; DiFranza et al. 2004); stronger antigen-induced lymphoproliferation in cord blood (Devereux et al. 2002); and significantly higher cord blood mononuclear cell production of Th2 cytokines (interleukin-13) in response to stimulation with antigen (Noakes et al. 2003).

This report extends earlier work on births in the Czech Republic. In contrast to earlier analyses of long-term ambient air pollution, in which we focused on the comparison of births from a highly versus a less polluted district (Hertz-Picciotto et al. 2002), in the present project we examine temporal variability in exposures in the days immediately before birth. In particular, we examine associations between neonatal immunophenotypes and specific pollutants not previously examined for developmental immunotoxicity—namely, PM_2.5_ (fine PM < 2.5 μm in aerodynamic diameter) and PAHs—in a large study population.

## Materials and Methods

### Subject enrollment and data collection.

From May 1994 through March 1999, women who delivered in the districts of Teplice or Prachatice were asked to participate in the Pregnancy Outcome Study (Dejmek et al. 2000). Of approximately 8,500 births in the two districts during the study period, nurses trained in research methods recruited close to 90% of mothers (*n* = 7,465) during their hospital stays and obtained written informed consent. While in the hospital, mothers completed self-administered questionnaires regarding reproductive histories, medical conditions and medications, smoking, alcohol and other lifestyle factors, and occupational information. The nurses then reviewed the mothers’ responses for ambiguity and completeness.

From this cohort, a subset of 1,476 mother–infant pairs was recruited into the Immune Biomarker Study, for which maternal and cord blood samples were obtained at delivery (Hertz-Picciotto et al. 2002). No exclusions were made, and a stratified random sample was obtained as follows: Nurses were instructed to enroll all preterm and low-birth-weight infants, as well as a systematic, one in five random sample of other births. However, sampling of the full-term, normal-birth-weight deliveries was increased when a meteorologic inversion occurred in January 1996 and was subsequently maintained through the end of the study. Friday or weekend deliveries were not enrolled because of the requirement for flow cytometry within 24 hr of sample collection.The overall sampling fraction was 20%. The refusal rate was 5%.

We abstracted data on pregnancy, labor, delivery, and the neonate from medical records—for example, birth weight, gestational age, date and time of birth, and medications used during each stage of labor. Home heating and cooking sources and family history of allergy were taken from a follow-up questionnaire administered to parents when children were 3 or 4.5 years of age. For the analysis below, we excluded data from *a*) mothers with incomplete sociodemographic or labor data (*n* = 36), *b*) infants delivered by cesarean section (*n* = 42), and *c*) one exceptionally long labor (> 24 hr), because labor itself is reported to influence lymphocyte distribution (Pittard et al. 1989), leaving 1,397 mother–infant pairs. This study was approved by the Ethical Committee of the Health Institute of Central Bohemia, Prague, Czech Republic, and the Institutional Review Board at the University of California, Davis.

### Exposure assessment.

In January 1992, an air-quality field measurement program was initiated by the Czech Ministry of Environment and carried out by the District Institutes of Hygiene, with assistance from the U.S. Environmental Protection Agency (Pinto et al. 1998). Two monitoring sites were established, one in Teplice, Northern Bohemia, and one in Prachatice, Southern Bohemia. Measurements of PM_2.5_, PM < 10 μm in diameter (PM_10_), and PAHs were performed daily in November–March, every third day in April–June and September–October, and every sixth day in July and August, when air pollution is lowest. Daily measurements of sulfur dioxide and nitrogen oxides were conducted year-round and were used in the imputation of PM_2.5_ and PAHs for nonsampled days.

We assessed fine and coarse particle concentrations, as well as PAH concentrations, from samples collected by the versatile air pollution sampler described by Pinto et al. (1998). This device is a modified dichotomous sampler, with a size-selective inlet of 10 μm aerodynamic diameter. A virtual impactor separates the airflow into two channels that collect fine particles (PM_2.5_) and a third that collects coarse particles (PM_2.5–10_). We determined the mass PM_2.5_ gravimetrically from a sample collected on a Teflon filter pack in the first fine-particle channel.

In the second fine-particle channel, a polyurethane foam (PUF) trap located downstream of a quartz filter collects PAHs that have either evaporated from the quartz filter or were originally in the gas phase. PAH concentrations are obtained by extraction of the PUF trap and the quartz filters, followed by high-performance liquid chromatography analysis. We measured 12 compounds and used their sum (total PAH) for analysis: phenanthrene, anthracene, fluoranthene, pyrene, benzo[*a*]anthracene, chrysene, benzo[*b*]fluoranthene, benzo[*k*]fluoranthene, BaP, dibenzo[*a*,*h*]anthracene, benzo[*g*,*h*,*i*]perylene, and indeno[*c*,*d*]pyrene. We calculated a BaP equivalent exposure using potency factors according to Petry at al. (1996). Seven PAHs potentially carcinogenic to humans were evaluated separately as the sum of c-PAHs: chrysene, benzo[*a*]anthracene, benzo[*b*]fluoranthene, benzo[*k*]fluoranthene, BaP, dibenz[*a,h*]-anthracene, and indeno[1,2,3-*c,d*]pyrene. This sum had been associated with intrauterine growth retardation in a prior analysis of the cohort (Dejmek et al. 2000).

For periods when daily PM_2.5_ and PAHs were not measured, between 3 and 5% of the values had to be imputed. The time-series nature of the data dictated an imputation procedure that replaced missing values with estimates that took into account the predictive capabilities of nearby neighbors in time as well as relations with other pollution series. To minimize the effects of potential outliers, the log-transformed values were imputed and then transformed back to determine imputed pollution values. The underlying pollution series were assumed to satisfy a first-order vector autoregressive model, with Kalman filters and smoothers (Shumway and Stoffer 2000) used for imputation. More specifically, the imputed values are always the conditional expectations of the unobserved values given the observed series. The general methodology follows that described in Little and Rubin (2002).

Because not all PAHs were detected each time measurements were made, we assumed that the log-transformed values were independent and normally distributed to complete the 12 dimensional component vectors. We imputed the missing observations on days when PAHs were measured (about 5.2% for Teplice and 3.8% for Prachatice) using conditional expectations obtained during the estimation process, following a commonly used procedure for estimating parameters with missing data (Little and Rubin 2002).

### Laboratory methods: flow cytometry.

Venous cord blood sampled immediately after labor was collected into heparinized Vacutainers (10-mL Vacuette; Greiner, Kremsmuenster, Austria). The samples were stored at 4°C in polystyrene boxes and transported for analysis in coolers. Samples arriving at the laboratory later than 24 hr after delivery were discarded; all others were analyzed on arrival. Lymphocytes in lysed whole blood were immunophenotyped using a FACSort flow cytometer, Simulset software, and a Simultest IMK lymphocyte kit of monoclonal antibodies (Becton Dickinson Immunocytometry Systems, San Jose, California, USA).

The following lymphocyte subsets were determined: CD3^+^ T-lymphocytes, CD3^−^CD19^+^ B-lymphocytes, and CD3^−^CD16^+^/CD56^+^ NK cells. Subsets of the CD3^+^ cells were also ascertained, including CD4^+^ and CD8^+^ cells, sometimes referred to as T-helper and T-suppressor cells, respectively. Problems with contamination of cord blood lymphocytes with nucleated red blood cells were solved by a lysed whole blood method (Harris et al. 1994). The Simulset software provides the three-part differential of leukocytes in the gate, based on CD45^+^CD14^+^ staining. Correspondence of the percentage of identified lymphocytes (sum of T + B + NK cells) to the percentage of lymphocytes in the gate was used to control the quality of staining. The proportions of the lymphocyte subsets were converted to percentages of the sum of T, B, and NK lymphocytes.

### Data handling and statistical methods.

We entered abstracted medical record data and maternal questionnaire information into electronic files in Prague at the Laboratory of Genetic Ecotoxicology (Institute of Experimental Medicine, Academy of Sciences, Czech Republic). One of us (M.D.) then reviewed the medical records a final time to correct errors. Outliers, implausible values, and inconsistencies across variables were identified and resolved. Multiple files were merged into an analysis file that included *a*) 24-hr average concentrations of air pollutants; *b*) daily mean temperature measurements; *c*) maternal, family, and household characteristics; *d*) data from medical records on pregnancy, labor, and delivery; and *e*) lymphocyte results.

To quantify the relationships between measurements of air pollutants and immune parameters, multiple linear regression models were fit separately for PM_2.5_ and total PAHs. For each of these, we examined the lymphocyte percentages in relation to exposures for five averaging periods before the date of cord blood collection: 3-, 7-, 14-, 30-, and 45-day intervals before birth. The strongest and most precise associations were with 7-day and 14-day intervals; for brevity, we present only the 14-day results.

Covariates of concern were identified from the literature, the conceptual model we developed *a priori*, and an empirical screen of the variables available. Empirically, we screened for predictivity using variables previously associated (*p* < 0.15) with lymphocyte distributions in cord blood (Hertz-Picciotto et al. 2002), including season, length of labor, parity, number of previous stillbirths, medication during delivery, working status of mother, maternal education, and exposure to active and secondhand smoke. In the present analysis, we also examined family history of allergy, self-reports of workplace exposure to dust during pregnancy, and self-reported maternal chronic or severe respiratory diseases during pregnancy.

Meteorologic variables are potential confounders because of strong associations with air pollution and lymphocytes (Afoke et al. 1993; Levi et al. 1988). Therefore, we explored five averaging periods of ambient temperature (3-, 7-, 14-, 30-, and 45-day intervals) for multivariable models. We show results adjusted for 3-day average temperature before birth to account for short-term and 45-day average temperature to represent potential longer term associations. In addition, seasonal and circadian rhythms are characteristics of both air pollutants and lymphocytes. We adjusted for time of day of delivery, as well as season, where summer (June–August) is the reference, with three binary variables representing winter (December–February), spring (March–May), and fall (September–November).

We evaluated four possible effect modifiers: home heating source, cigarette smoking, ethnicity, and low birth weight or prematurity. Coal or wood heating or cooking can markedly increase residential indoor exposure to PAHs (Siwinska et al. 1999), and because the effect of ambient air pollution might differ among those already exposed to high background exposures in their homes, we introduced an interaction term in the models. For similar reasons, we evaluated effect modification from cigarette smoking, either by the mother or by other members of the family. We also addressed possible heterogeneity by ethnicity—that is, whether associations with ambient air pollutants differed between newborns of Gypsy (Rom) ethnicity and those of European origin. As reported previously, frequency of smoking was greater among the Roma, parity was higher, and infants were more likely to be low birth weight. Finally, infants born prematurely or who were small for their gestational age were hypothesized to represent a potentially susceptible subgroup.

The initial model included average PAH level during the 14 days before birth and all variables on the candidate list that were appropriate for adjustment. Subsequently, predictor variables were removed if they did not predict the outcome with adequate precision (i.e., they were eliminated if *p* > 0.15), did not block a backdoor path in the directed acyclic graph (Cole and Hernan 2002), and resulted in changes < 15% in the estimated coefficient for PAHs. Once the predictive or confounder covariates were determined, they were used in subsequent models exploring different averaging periods or alternative pollutant mixes (i.e., BaP equivalents or carcinogenic PAHs). To ensure that confounders specific to the other pollutant groups were not missed, the final models for PM_2.5_, BaP equivalents, and carcinogenic PAHs were expanded to examine potential confounding (by the change-inestimate criterion) from any previously removed variables.

All models were adjusted using SUDAAN statistical software (version 8.2004; Research Triangle Institute, Research Triangle Park, NC, USA) for the sampling design, namely, stratified sampling without replacement in strata defined by three variables (district, preterm or low birth weight vs. full term and normal birth weight, and year of birth). Results were expressed as predicted changes in lymphocyte distribution for an increase of 100 ng/m^3^ in PAH concentration, and of 25 μg/m^3^ in PM_2.5_. These increments were close to two standard deviations of the distributions of 14-day averages of the two pollutants in this study: 48 ng/m^3^ for total PAHs and 13 μg/m^3^ for PM_2.5_. We chose to use absolute increments, rather than interquartile ranges, to facilitate comparison across studies.

## Results

Table 1 compares the 1,397 deliveries in the immunity study that met our inclusion criteria with the full cohort from which they were sampled. The sample in the immunity study consisted of proportionally fewer births from Teplice (28% vs. 39% from Prachatice), more mothers of low parity, and, because of the sampling strategy, more winter/spring and 1996–1998 births and more preterm and low-birth-weight infants. The sample did not differ from the full cohort with respect to maternal or paternal education, smoking, maternal age, or ethnicity.

**Figure 1:**
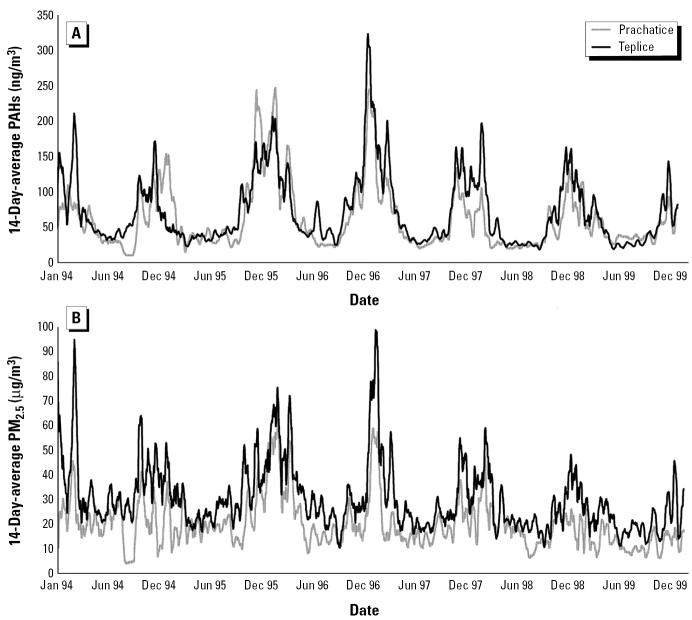
Time series plots for the two districts in the study. (A) PAHs. (B) PM_2.5_.

Mean daily averages and mean 14-day averages were nearly identical both within districts and averaged across districts (e.g., in Teplice the daily mean PAH concentration was 68.7 ng/m3, whereas the 14-day mean was 68.9 ng/m3). PAHs were about 10% lower in Prachatice. However, Prachatice PAH concentrations were higher in the winters of 1994–1995 and 1995–1996 (Figure 1). The interdistrict differences are more pronounced for PM_2.5_: The mean 14-day average over the study period was about 1.5 times higher in Teplice (30.1 μg/m3) than in Prachatice (19.8 μg/m3). These PM_2.5_ differences are fairly consistent throughout the study period, with close tracking of seasonal peaks in the two districts. Correlations varied by district (Table 2). Temperature showed strong negative correlations with both PAHs (r = −0.7 to −0.9) and PM_2.5_ (r = −0.4 to −0.6). The correlation of 14-day average PAHs with PM_2.5_ was 0.6 in Prachatice and 0.9 in Teplice. When PAHs from different averaging periods were compared, the correlations declined from almost 1 (3-day vs. 7-day average) to 0.9 (3-day vs. 45-day average); for PM_2.5_, these correlations ranged from a high of 0.9 to 0.5 (data not shown).

**Figure 2:**
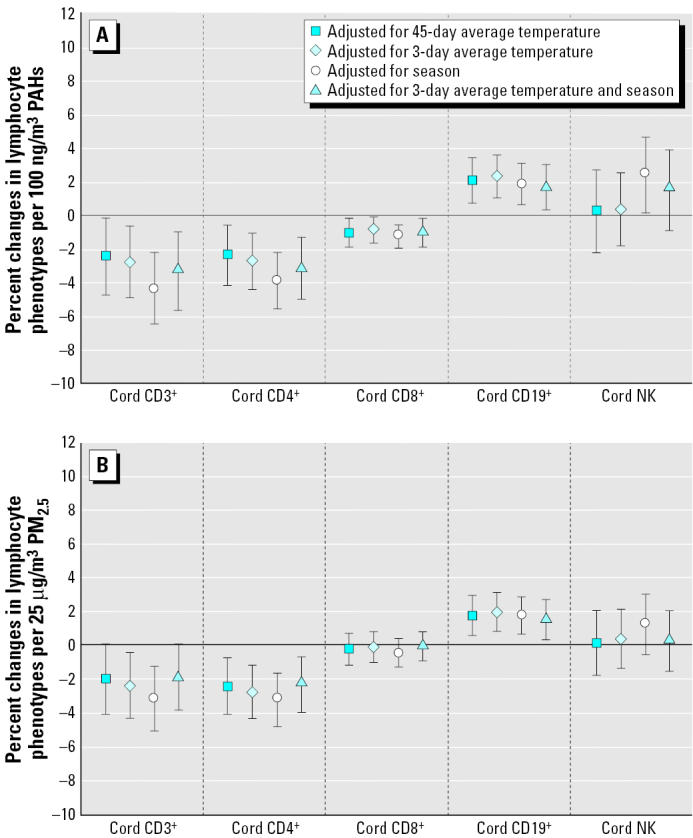
Associations between air pollutants and lymphocyte fractions: Percent changes in cord blood lymphocyte distributions, with 95% CIs, associated with increases of 100 ng/m^3^ PAH (A) or 25 μg/m^3^ PM_2.5_ (B) during the 14 days before birth. All models adjusted for district, year of birth, time of day of delivery, labor medication and duration, number of previous pregnancies, and maternal education and smoking.

Adjusted for 3-day temperature and season, total PAH exposure during the 14 days before birth was associated with reduced T-lymphocyte fractions CD3+, CD4+, and CD8+, and an increase in the B-lymphocyte fraction (CD19+) (Figure 2). For a 100-ng/m3 increase in PAHs, the percentage decrease was −3.3% [95% confidence interval (CI), −5.6 to −1.0%] for CD3+, −3.1% (95% CI, −4.9 to −1.3%) for CD4+, and −1.0% (95% CI, −1.8 to −0.2%) for CD8+ cells. The corresponding increase in the CD19+ cells was +1.7% (95% CI, 0.4 to 3.0%).

These findings were robust to the parameterization of temperature and season; the strongest findings for all fractions, except CD19+, were observed when adjustment was made for season alone. In the models adjusted for season, an increase of the NK-cell fraction was also seen (+2.5%; 95% CI, 0.2 to 4.7%); additional adjustment for 3-day temperature reduced the magnitude of associations, but most remained significant (Table 3).

These findings were robust to the parameterization of temperature and season; the strongest findings for all fractions, except CD19+, were observed when adjustment was made for season alone. In the models adjusted for season, an increase of the NK-cell fraction was also seen (+2.5%; 95% CI, 0.2 to 4.7%); additional adjustment for 3-day temperature reduced the magnitude of associations, but most remained significant (Table 3).

When the alternative PAH averaging periods (3, 7, 30, and 45 days) were used, the results usually yielded lower precision, although the overall patterns were fairly similar (data not shown); for models using 7-day averages, the results were very close to those from the 14-day models—that is, none of the associations with lymphocyte subsets changed by > 15%.

Models using PM_2.5_ concentrations showed similar results for CD4+, CD3+, and CD19+, whereas associations for CD8+ disappeared. Associations were very much attenuated or not present in models of BaP equivalents or carcinogenic PAHs (data not shown).

Temperature (either 3- or 45-day average before birth) was a strong predictor of all lymphocyte phenotype subsets except B-cells (CD19+). When 3-day temperature was included in the model, fall season was strongly related to increased CD3+ (+3.7%; 95% CI, 1.0 to 6.4%) and CD4+ cell fractions (+3.7%; 95% CI, 1.4 to 6.1%) and a lower CD19+ cell fraction (−2.6%; 95% CI, −4.1 to −1.0%). Spring was associated with lower CD19+ (−1.9%; 95% CI, −3.6 to −0.1%) and higher NK (+2.8%; 95% CI, 2.7 to 2.9%) cell fractions.

Other variables predictive of one or more lymphocyte immunophenotypes were district and year of birth, duration of labor, medications administered during labor, number of previous pregnancies, maternal education, time of day of delivery, and workplace exposure to dust. Family history of allergy and maternal chronic respiratory diseases during pregnancy were not associated with lymphocyte phenotype fractions in cord blood and were therefore not included in the final models.

**Figure 3:**
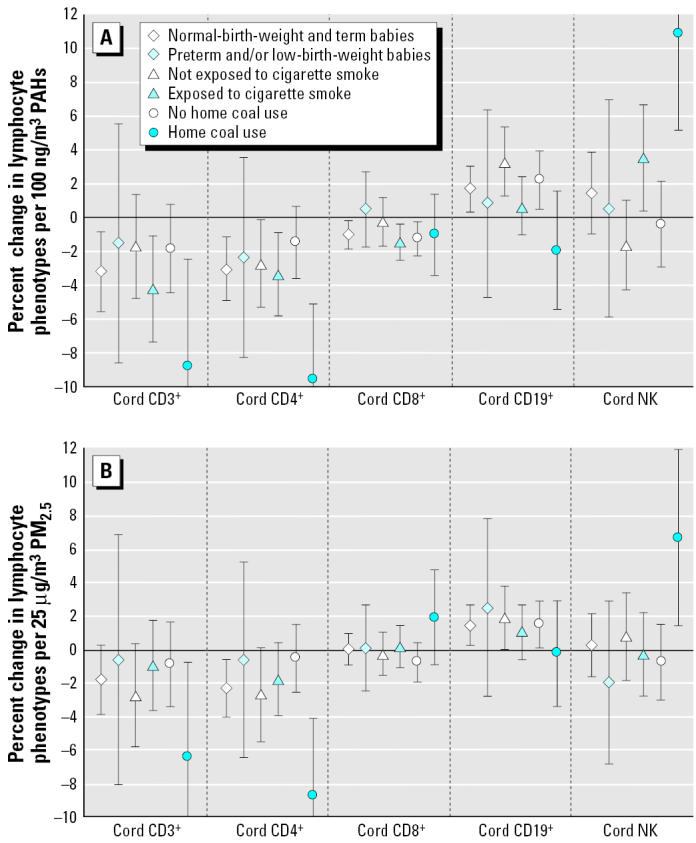
Effect modification of associations between air pollutants and lymphocyte fractions by size and maturity at birth, exposure to cigarette smoke and home coal use: Percent changes in cord blood lymphocyte distributions, with 95% CIs, associated with increases of 100 ng/m^3^ PAH (A) or 25 μg/m^3^ PM_2.5_ (B) during the 14 days before birth, in various subsets of the study population. All models adjusted for season, average temperature in the three days before birth, district, year of birth, time of day of delivery, labor medication and duration, number of previous pregnancies, and maternal education and smoking.

We observed no difference in the impact of any of the air pollutants between the Roma infants and infants born to mothers of eastern European descent (data not shown). Among newborns from homes using coal or wood for cooking or heating, PAHs were associated with greater decreases in fractions of CD3+ and CD4+ and greater increases in the percentage of NK cells (Figure 3). The pollutant-associated decreases of CD3+, CD4+, and CD8+ percentages were greater in newborns exposed to cigarette smoke from the mother or others around her versus newborns not exposed to cigarette smoke, whereas an increase in the CD19+ percentage was observed only among births to nonsmokers. PAH-associated increases in NK cell fractions were largest in homes of coal users and smokers. For PM_2.5_, the only demonstrable heterogeneity was observed for neonates in homes using coal, with greater reductions in CD3+ and CD4+ percentages and an elevated percentage of NK cells, a pattern similar to that observed in relation to ambient PAHs.

Preterm or low-birth-weight infants were not at higher risk than were normal neonates for pollution-associated changes in lymphocyte proportions (Figure 3). However, in this subset, there appeared to be an interaction between ambient PAH exposure and maternal exposure (active or passive) to cigarette smoke (not shown in figure). Specifically, preterm/low-birth-weight infants born to mothers reporting active or second-hand cigarette smoke exposure had cord blood samples with a decreased proportion of cells with CD3+ surface antigens (−9.7%; 95% CI, −15.0 to −4.4%), whereas cells with CD19+ were elevated by 3.8% (95% CI, 0.3 to 7.3%) and NK cells were elevated by 4.5% (95% CI, 0.6 to 8.4%), for an increase of 100 ng PAH/m3, compared with preterm/low-birth-weight infants born to mothers not exposed to cigarette smoke.

## Discussion

In this study population, ambient concentrations of PAHs and PM_2.5_ during the last 2 weeks of gestation were associated with decreases in the percentages of T-lymphocytes in cord blood. These associations were stronger for the percentage of CD4+ cells than for the percentage of CD8+ subsets. However, partly because CD4+ are relatively more numerous, the resulting effect of air pollution on the CD4+:CD8+ ratio was essentially null. The association with CD8+ cells was more marked for PAHs, compared with PM_2.5_. Accompanying these T-cell decreases, the percentage of B-cells (CD19+) increased. Because these outcomes represent distributions of immune phenotype subsets relative to each other, if the percentage of one subset goes down, the percentage of at least one other will increase. It would have been informative to measure absolute lymphocyte counts and functional immune parameters; however, because the quality of these measurements degrades with increasing storage time and transportation, such measurements were deemed not feasible in this project.

The possibility that redistributions of lymphocyte phenotypes may alter susceptibility to infections or inflammatory diseases in otherwise healthy persons, particularly small children, is worthy of investigation in its own right. The longitudinal life-course changes in lymphocyte populations, both absolute and relative, are discussed by Schultz et al. (2000), who concluded that the developmental patterns in absolute counts were different from those in relative distributions, and that “discordance between the absolute and relative size of lymphocyte subpopulations emphasizes the consideration of both variables in the assessment of lymphocyte maturation.” Published data suggest that the percentages of many of the immunophenotypic subpopulations differ for cord versus adult blood (Zhao et al. 2002) but that differences in absolute counts are not always accompanied by differences in percentages (D’Arena et al. 1998). Percentages of some lymphocyte subtypes change from the fetal to neonatal period (Zhao et al. 2002). Given that immunologic development is intimately connected to the interactions between the organism and the environment via antigenic challenge, specific chemical exposures could influence these relationships (e.g., exposure to diesel particles can modify the host response to allergen) (Diaz-Sanchez et al. 2003). Whether alterations in developmental patterns of lymphocytes have clinical implications remains to be established. To shed light on the larger picture, we are addressing, in other work, immunoglobulin production of the neonate and relationships between relative lymphocyte distributions at birth and subsequent early childhood morbidity.

The direction of associations was generally unchanged, whether adjusted for short-term (3 days) or longer-term (45 days) average temperature, season, or both season and short-term temperature, although the precision and magnitude varied. The associations with CD19+ were the most consistent across pollutant metrics (Table 3).

Few studies have examined the relationship of air pollution to pediatric immune status. Leonardi et al. (2000) examined absolute levels of specific lymphocytes in schoolchildren, whereas we assessed percentages of lymphocytes in newborns; hence, our results cannot be compared. Similarly, a cross-sectional study of schoolchildren in two industrial cities suggested a relationship of air pollution with changes in several immune cell fractions, but this report was based on ecologic, not individual-level, data and did not control for any confounders (Skachkova et al. 2001). Overall, the proportions of T-, B-, and NK lymphocytes in cord blood in our study were not markedly different from those reported in a small Italian study (D’Arena et al. 1998) and a large Mexican sample (Garcia et al. 1995). Different laboratory methods and our inclusion of a population-based sample, with no exclusions, can explain any discrepancies.

Consistent with previous findings in this project (Hertz-Picciotto et al. 2002) and other studies, the length of labor, delivery medication, number of previous pregnancies, maternal education, and time of day of delivery (Levi et al. 1988) were all related to lymphocyte outcomes at birth. Maternal chronic or severe respiratory diseases during pregnancy did not predict lymphocyte distributions at birth, possibly because these self-reports did not include information on the period in gestation during which they occurred. Although our previous findings using district of residence as a surrogate for chronic exposure might have been confounded by unrecognized interdistrict differences (Hertz-Picciotto et al. 2002), this problem was eliminated by analysis of short-term temporal variations in PM_2.5_ and PAHs before birth, in which district and other confounders were controlled. The PM_2.5_ fraction was selected because it generally displays less spatial heterogeneity than do coarse particles. With only one monitor in each district, fine particles would be subject to less misclassification error. Nevertheless, some error is unavoidable, especially for families living at some distance from the monitors.

Participants in the immune biomarker study were randomly sampled as described above, and the refusal rate was < 5%, supporting the validity of the results based on this sample. Although the distributions of several variables were significantly different in the study sample compared with the full cohort (e.g., the proportion of low-birth-weight infants was 7% in the study sample and 5% in the full cohort), these differences are not likely to have affected either the internal or external validity of the results, because the variables were controlled in the analysis through adjustment for the sampling design or by multivariate adjustment or both. These adjustments combined with the use of sampling weights also provided generalizability to the population that participated in the full cohort study—about 90% of the population in the two districts. Because 14 days before birth could represent different developmental periods for low-birth-weight and preterm newborns, we also conducted a sensitivity analysis in which these infants were excluded and found little change in the results (data not shown).

We furthermore believe our results could be generalized outside the Czech Republic, given that the exposures are similar to those in other countries. Concentrations of PAHs in this investigation were in a range similar to what has been reported from some U.S., European, and Asian cities. For instance, for BaP, our geometric mean was 1.4 ng/m3, which was comparable to levels observed in Pavia (Italy) in 1996 and in Taipei in 1995 and 1996, 1.2 and 1.7 ng/m3, respectively (Naumova et al. 2002).

In the Central European Study of Air Quality and Respiratory Health (CESAR, conducted in Bulgaria, Hungary, Czech Republic, Slovak Republic, Poland, and Romania) annual PM_2.5_ means were between 29 and 67 μg/m3 (Leonardi et al. 2000). Although the annual average concentration in our study was lower (25 μg/m3), the winter mean was 38 μg/m3, which is higher than the winter means of 18 of the 21 cities analyzed in the European Community Respiratory Health Survey study (Hazenkamp-von Arx et al. 2003). Most U.S. cities are reported to have lower annual PM_2.5_ means, although comparable means occurred in Los Angeles (25 μg/m3) and Riverside (29 μg/m3), California, in 2000 (Pinto et al. 2004).

The less than daily monitoring during 7 months of the year made it necessary to impute pollution data. Given the schedule of every third day of measurement for 5 months of the year, and every sixth day for 2 months, the probability that missing data are related to levels of PM_2.5_ or PAH, after conditioning on daily measurements for SO_2_ and NO_x_ and on the existing data for PM_10_, PM_2.5_, and PAH, appears to be extremely small. Thus, the rationale for the assumption of “missing-at-random” is strong.

Confounding due to meteorology and seasonal lymphocyte patterns was accounted for by including 3- or 45-day averages of temperature or season, or both season and temperature in the model. Short-term and longer term time spans of antenatal ambient temperatures were related to a similar extent to T-lymphocyte fractions. Except for CD19+, inclusion of either temperature interval (3- or 45-day average before birth) resulted in less precise estimates of the pollution effects. Season alone does not appear to have this effect; winter by itself does not have any impact once temperature is included. Possibly, our adjustment for temperature could induce bias, because it is highly correlated (Table 2) with air pollution, particularly within season; however, the direction of such bias is difficult to deduce.

The B-lymphocyte fraction was not related to ambient temperature but was related to both spring and fall seasons. This suggests that the seasonal associations must be due to other variables, perhaps respiratory infections during fall or pollen in spring. Seasonal variations in immunologic parameters occur in healthy children (Afoke et al. 1993) and are hypothesized to represent adaptive responses to climatic variability and other environmental factors.

We also found that maternal active smoking and/or exposure to second-hand smoke predicted cord blood lymphocyte distributions in preterm low-birth-weight newborns (decreased T-lymphocytes and increased B-lymphocytes). We did not find published results on lymphocyte phenotype fractions in cord blood in relation to cigarette smoking. Studies in adults consistently suggest a rise in either the percentage or absolute count of CD3+ or CD4+ cells in association with cigarette smoke exposure (Santagostino et al. 1999; Schaberg et al. 1997; Tollerud et al. 1989). One of these studies also reports a higher B-cell fraction related to smoking (Santagostino et al. 1999), which is concordant with our finding.

The pattern in relation to cigarette smoke in our data was similar to what we observed for lymphocyte distributions in relation to ambient air pollution, that is, PAHs. Whether this similarity is due to PAHs or other constituents common to tobacco smoke and ambient air pollution is unclear. Furthermore, lymphocyte changes associated with ambient PAHs in all but the B-cell fractions were greater in births where the mother or others around her smoked (Figure 3). This association of PAHs beyond the effect of smoke exposure on lymphocyte phenotype fractions is consistent with an impact of these aromatic compounds over a wide range of exposure levels.

The subgroup of children most susceptible to lymphocyte effects from air pollution appears to be those from homes heated by coal. This may be a result of high exposures to PAHs from this source.

Distinct from our earlier work suggesting that chronic, long-term exposure to high ambient air pollution may influence immune development (Hertz-Picciott et al. 2002), these results demonstrate short-term associations with fine particles and PAHs. Adjustment for temperature does not eliminate these associations. A comparison of the associations with PAHs and with PM_2.5_ indicates a number of stronger findings for the former. Reductions in CD3+ and CD4+ fractions were larger for PAHs, and no association was observed between PM_2.5_ and CD8+ fractions. Because the increments used for PAHs and PM_2.5_ represented approximately a change of two standard deviations for each, these results can be compared.

Our measurements included both the semivolatile and particulate-bound fractions of PAHs. Given this detailed characterization of PAHs and the associations observed here, chemical composition may play a key role in the immune-mediated effects of air pollution. Others have demonstrated that fetal exposures to ambient PAHs occur trans-placentally and can result in mutations in cord blood lymphocytes (Perera et al. 2002). Moreover, PAHs have been demonstrated to exhibit immunomodulatory properties (Nel et al. 2001).

The striking finding of this study is that exposure to ambient PM_2.5_ and PAHs in late pregnancy is associated with statistically significant changes in the distributions of lymphocyte phenotypes in cord blood. Although the biologic relevance of this finding is not entirely clear, the observation of note is that the fetal immune system may be altered by maternal exposure to these environmental pollutants. Further research on critical windows of vulnerability throughout gestation is warranted, as well as study of whether such changes persist beyond birth and/or are associated with adverse health effects. We have recently completed a follow-up study of these children, which will give the opportunity to relate the changes observed in T-cell and B-cell fractions at birth with subsequent morbidity.

## Figures and Tables

**Table 1:** Comparison of characteristics at birth in full cohort versus subset with lymphocytes in cord blood: deliveries in 1994–1999, Prachatice and Teplice, Czech Republic.

Characteristic	Immunity [*n* = 1,397; no. (%)]	Full cohort [*n* = 7,502; no. (%)]
District*		
Prachatice	548 (39)	2,144 (29)
Teplice	849 (61)	5,358 (71)
Season of birth*		
Winter	380 (27)	1,826 (24)
Spring	397 (28)	2,017 (27)
Summer	300 (21)	1,929 (26)
Fall	320 (23)	1,730 (23)
Year of birth*		
1994	106 (8)	1,313 (18)
1995	181 (13)	1,606 (21)
1996	300 (22)	1,420 (19)
1997	367 (26)	1,419 (19)
1998	374 (27)	1,394 (19)
1999	69 (5)	350 (5)
Delivery hour		
0600–1159 hr	383 (28)	2,082 (28)
1200–1759 hr	402 (29)	2,101 (28)
1800–2359 hr	316 (23)	1,668 (22)
0000–0559 hr	285 (20)	1,583 (21)
Missing	11 (1)	68 (1)
Sex		
Male	724 (52)	3,856 (51)
Female	673 (48)	3,643 (49)
Missing	0 (0)	3 (0)
Birth weight (g)*		
≤ 2,500	102 (7)	365 (5)
> 2,500	1,294 (93)	7,132 (95)
Missing	1 (0)	5 (0)
Weeks of gestation at birth*		
< 37	98 (7)	339 (5)
≥ 37	1,299 (93)	7,163 (96)
Mother’s age at delivery (years)		
< 20	171 (12)	934 (13)
20–24.9	638 (46)	3,274 (44)
25–29.9	395 (28)	2,120 (28)
30–34.9	142 (10)	856 (11)
≥ 35	51 (4)	313 (4)
Missing	0 (0)	5 (0)
Ethnicity of mother		
European	1,228 (88)	6,556 (87)
Romani	155 (11)	856 (11)
Other	9 (1)	70 (1)
Don’t know	2 (0)	14 (0)
Missing	3 (0)	6 (0)
No. of live births (parity)*		
0–1	651 (47)	2,813 (38)
2	519 (37)	2,306 (31)
≥ 3	227 (16)	2,348 (31)
Missing	0 (0)	35 (0)
Mother’s education		
Did not complete primary school	18 (1)	120 (2)
Primary school	293 (2)	1,587 (21)
Secondary school	602 (43)	3,205 (43)
Secondary school with leaving exam	400 (29)	2,092 (28)
Student	6 (0)	36 (0)
University	78 (6)	404 (5)
Missing	0 (0)	58 (1)
No. of cigarettes/day smoked by mother before pregnancy		
None	880 (63)	4,584 (61)
1–10	286 (21)	1,593 (21)
11–20	186 (13)	1,033 (14)
≥ 21	20 (1)	130 (2)
Missing	25 (2)	162 (2)
No. of cigarettes/day smoked by father during pregnancy		
None	627 (45)	3,197 (43)
1–10	312 (22)	1,670 (22)
11–20	365 (26)	2,004 (27)
≥ 21	42 (3)	310 (4)
Missing	51 (4)	321 (4)
Father’s education		
Did not complete primary school	10 (1)	53 (1)
Primary school	207 (15)	1,250 (17)
Secondary school	722 (52)	3,757 (50)
Secondary school with leaving exam	305 (22)	1,593 (21)
Student	5 (0)	28 (0)
University	91 (7)	443 (6)
Missing	57 (4)	378 (5)

* Chi-squared, p < 0.05.

**Table 2:** Means and Spearman correlations for pollutants and temperature, April 1994 through March 1999 (n = 1,796 days).

	Arithmetic mean	14-Day PAH	14-Day PM_2.5_	3-Day temperature	45-Day temperature
Prachatice					
14-Day PAH (ng/m3)	62.77	1.00	0.56	−0.67	−0.70
14-Day PM_2.5_ (ng/m3)	18.49		1.00	−0.45	−0.47
3-Day temperature (°C)	7.58			1.00	0.83
45-Day temperature (°C)	7.61				1.00
Teplice					
14-Day PAH (ng/m3)	70.00	1.00	0.79	−0.80	−0.75
14-Day PM_2.5_ (ng/m3)	28.80		1.00	−0.54	−0.58
3-Day temperature (°C)	9.54			1.00	0.86
45-Day temperature (°C)	9.52				1.00

All correlations are significant at p < 0.0001.

**Table 3:** Adjusted^a^ percent changes in cord lymphocyte outcome for increments of 100 ng/m^3^ PAH and 25 μg/m^3^ PM_2.5_ average during 14 days before birth: impact of adjustment for meteorologic variables.

		Models adjusted for the following meteorologic variables							
		45-Day average temperature		3-Day average temperature		Season		3-Day average temperature + season	
Lymphocyte	Air pollution	Percent change (95% CI)	p-Value	Percent change (95% CI)	p-Value	Percent change (95% CI)	p-Value	Percent change (95% CI)	p-Value
CD3	100 ng/m3 PAHs	−2.43 (−4.74 to −0.12)	0.04	−2.74 (−4.81 to −0.66)	0.01	−4.33 (−6.44 to −2.22)	< 0.0005	−3.26 (−5.55 to −0.98)	0.01
	25 μg/m3 PM_2.5_	−1.95 (−4.00 to 0.10)	0.06	−2.34 (−4.29 to −0.39)	0.02	−3.12 (−5.05 to −1.19)	< 0.0005	−1.86 (−3.81 to 0.09)	0.06
CD4	100 ng/m3 PAHs	−2.33 (−4.12 to −0.53)	0.01	−2.68 (−4.34 to −1.03)	< 0.0005	−3.86 (−5.54 to −2.18)	< 0.0005	−3.12 (−4.94 to −1.29)	0.0005
	25 μg/m3 PM_2.5_	−2.37 (−4.04 to −0.69)	0.01	−2.71 (−4.35 to −1.07)	< 0.0005	−3.18 (−4.79 to −1.56)	< 0.0005	−2.27 (−3.93 to −0.60)	0.01
CD8	100 ng/m3 PAHs	−1.01 (−1.85 to −0.18)	0.02	−0.80 (−1.58 to −0.03)	0.04	−1.23 (−1.96 to −0.51)	< 0.0005	−1.01 (−1.82 to −0.19)	0.02
	25 μg/m3 PM_2.5_	−0.16 (−1.08 to 0.75)	0.73	−0.07 (−0.93 to 0.80)	0.88	−0.39 (−1.21 to 0.43)	0.35	−0.02 (−0.89 to 0.85)	0.96
CD4:CD8	100 ng/m3 PAHs	0.21 (−0.08 to 0.49)	0.15	0.16 (−0.19 to 0.32)	0.27	0.07 (−0.19 to 0.32)	0.61	0.11 (−0.18 to 0.40)	0.46
	25 μg/m3 PM_2.5_	0.09 (−0.20 to 0.37)	0.55	0.05 (−0.25 to 0.35)	0.73	0.03 (−0.24 to 0.29)	0.83	0.06 (−0.24 to 0.36)	0.72
CD19	100 ng/m3 PAHs	2.11 (0.74 to 3.45)	0.003	2.34 (1.04 to 3.63)	< 0.0005	1.87 (0.65 to 3.08)	0.003	1.69 (0.36 to 3.01)	0.01
	25 μg/m3 PM_2.5_	1.77 (0.56 to 2.98)	< 0.0005	1.96 (0.79 to 3.12)	< 0.0005	1.81 (0.70 to 2.92)	< 0.0005	1.55 (0.39 to 2.71)	0.01
NK	100 ng/m3 PAHs	0.27 (−2.18 to 2.71)	0.83	0.39 (−1.77 to 2.55)	0.72	2.45 (0.22 to 4.68)	0.03	1.54 (−0.83 to 3.92)	0.20
	25 μg/m3 PM_2.5_	0.13 (−1.76 to 2.02)	0.89	0.37 (−1.39 to 2.13)	0.68	1.28 (−0.54 to 3.10)	0.17	0.27 (−1.53 to 2.06)	0.77

a Adjusted for district, year and time of birth, labor medication and duration, number of previous pregnancies, maternal education, and maternal active smoking or exposure to second-hand smoke.
